# Poisoning suicide due to paroxetine overdose toxicity aided by benzodiazepine and antipsychotic drugs

**DOI:** 10.1007/s00414-025-03570-7

**Published:** 2025-08-04

**Authors:** Dimitra Florou, Research Fellow, Kleio-Evangelia Fragkouli, Amvrosios Orfanidis, Vassiliki A. Boumba

**Affiliations:** Department of Forensic Medicine & Toxicology, Faculty of Medicine, School of Health Sciences, University of Ioannina, Ioannina, 45500 Greece

**Keywords:** Paroxetine, Suicide, Alprazolam, Clozapine, Quetiapine, ICD-10

## Abstract

This contribution presents a case of suicide death due to ingestion of paroxetine in overdose by a 22-year-old psychiatric patient. The deceased was found dead in his room with blood exiting his nose and dry bloodstains on his left wrist. Many empty and semi-empty packets of prescribed medications were found in the scene. On internal examination, the urinary bladder was markedly distended, and the organs showed autolytic changes with no other macroscopic pathological lesions. Blood (from the left external iliac vein), urine, and gastric contents were sampled for toxicological analysis. The toxicological screening revealed the presence of the antidepressant selective serotonin reuptake inhibitor (SSRI) paroxetine, the benzodiazepine alprazolam and its metabolite alpha-hydroxy-alprazolam, and the antipsychotic quetiapine in blood, urine, and gastric contents, and the presence of nor-clozapine in urine. Quantitative analysis showed lethal blood paroxetine concentration of 3.3 mg/L and 1.58 mg/L in urine. Based on the medicolegal autopsy, the medical history, the circumstances of death by the police, the toxicological results and the pharmacology of the involved drugs, the manner and cause of death were recorded as suicide and, mixed-drug intoxication due to the synergistic action of the primary toxicant paroxetine and the secondary contribution of alprazolam, quetiapine and clozapine, respectively. It was classified as X61 in International Classification of Diseases (10th revision). This is the first fatal case reporting the simultaneous presence of antipsychotics (quetiapine and clozapine) and benzodiazepines (alprazolam) with toxic paroxetine levels.

## Introduction

Paroxetine is a selective serotonin reuptake inhibitor (SSRI) commonly prescribed for depression, anxiety disorders, and other mental health conditions. It is widely regarded as the most effective SSRI currently available for clinical application and it is considered a relatively safe antidepressant drug in respect to overdose. Paroxetine is highly potent and selective in inhibiting serotonin reuptake, with minimal effects on other neurotransmitters [1, 2]. Paroxetine exhibits a high degree of plasma protein binding, with around 95% of the drug being bound [1]. Its metabolism occurs mainly in the liver and is predominantly facilitated by the cytochrome enzyme CYP2D6, with contributions from CYP3A4 and potentially other cytochrome enzymes [1]. The pharmacokinetics of this medicine may be affected by genetic variations in the CYP2D6 enzyme [1–4]. It demonstrates a noteworthy lack of potentially fatal adverse effects [3, 4], such as cardiac and central nervous system (CNS) harm, primarily due to restricted receptor antagonism. While generally safe when used as prescribed, overdose can lead to severe symptoms and potential life-threatening complications [1–5]. More specifically, SSRIs could cause QT interval prolongation, which is usually associated with a ventricular arrhythmia that can result to sudden cardiac death [6]. Additionally, SSRIs could cause a serious complication known as serotonin syndrome [7]. Serotonin syndrome is a clinical condition, presenting as a serious complication of treatment with SSRIs and is generally characterized by the sudden onset of behavioral changes, neuromuscular shifts and autonomic dysfunctions. The diagnosis is made on a clinical basis and there are no specific pathognomonic features or laboratory findings that characterize it. It is worth mentioning that in previous fatalities due to paroxetine overdose it has been suggested as the mechanism of death [8–10]. Moreover, another possible mechanism of paroxetine toxicity could be the ventricular arrhythmia exerting complex vasoactive effects on the vascular smooth muscle [10].

It is accepted that postmortem peripheral blood paroxetine concentrations higher than 0.4 µg/mL could lead to a fatal outcome [10], while concentrations ranging from 1.4 to 4.0 mg/L are usually reported for fatal overdoses [9, 11, 12]. On the other hand, serum paroxetine concentrations of 1.9 mg/L and 1.8 mg/L were detected in acutely poisoned adults who ingested 2 g and 4 g paroxetine, respectively [13]. Obviously, there is considerable interindividual variation in the assessment and toxicity of paroxetine, where CYP2D61 polymorphisms have been suggested as main contributors [14]. In patients taking multiple drugs that cause central nervous system- and/or respiratory-depressant effects (such as neuroleptics, benzodiazepines, SSRIs) they may be additively or synergistically increased leading to adverse outcomes [15].

As regards suicide, it is strongly associated with psychiatric disorders and is particularly likely among adolescents and young adults [16]. Across the globe, suicide ranks as the second most common cause of premature death for young adults [17] while, SSRIs are included among the drugs used for deliberate drug overdose [18].

This contribution presents a case of suicide death of a 22-year-old psychiatric patient due to ingestion of paroxetine in overdose; the autopsy and toxicology findings are presented, compared to previously reported data in the literature and thoroughly discussed regarding the possible mechanism of toxicity and death.

## Methodology

### Case background

**Fig. 1:**
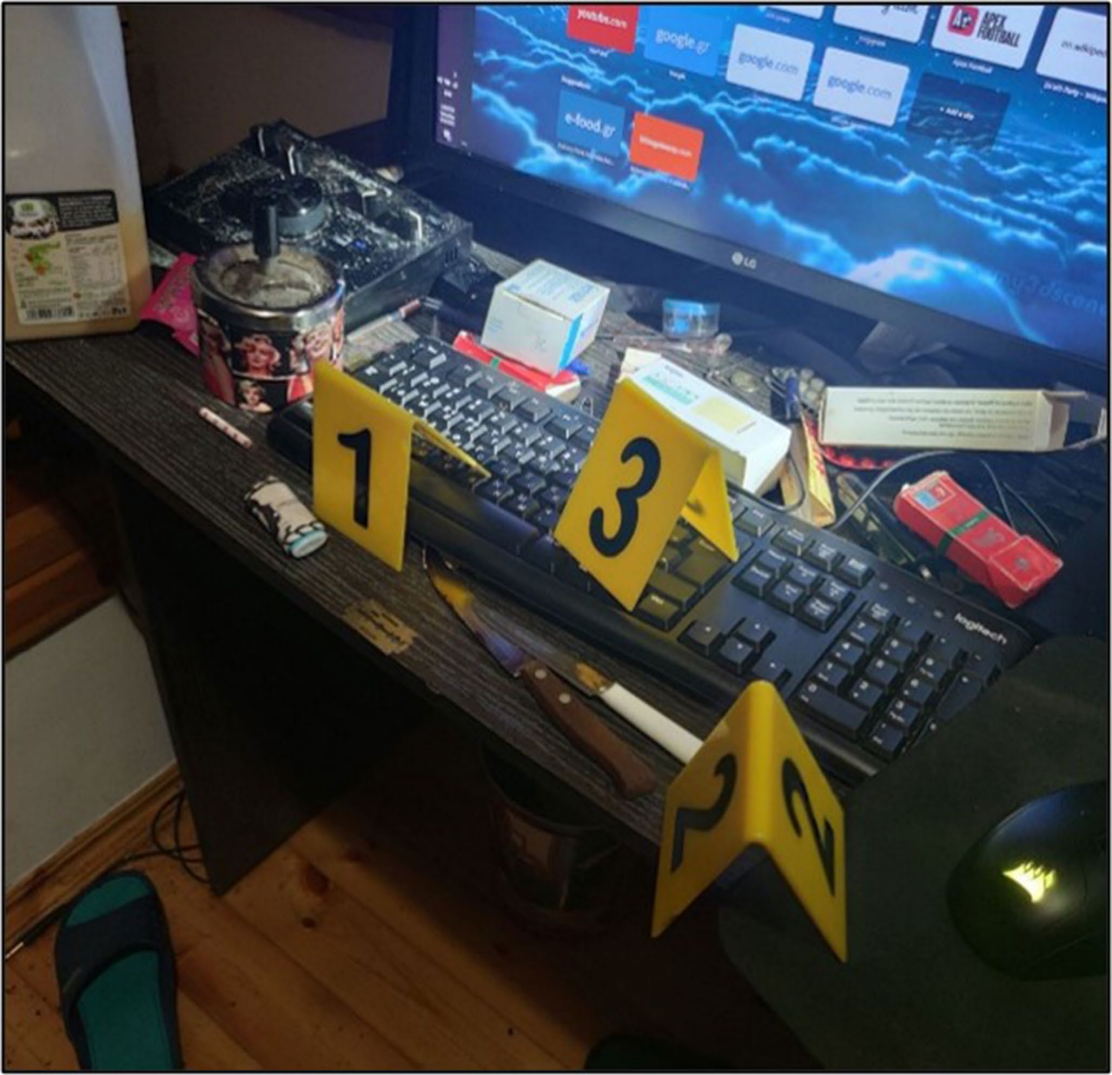
Empty packets of medications scattered on the desk in decedent’s room. A razor was also present at the lower edge of the desk

Α 22-year-old obese man was found dead in his room in early autumn. His body was discovered on the floor beside his desk, in a prone position, with blood exiting his nose and dried bloodstains present on his left wrist. Many empty packets of medications were scattered across the floor, on the desk and in a bin, close to the location of the body (Fig. 1). These packets were identified as his prescribed medications, and included paroxetine, quetiapine and alprazolam. The deceased had a history of major depression and panic disorder and was undergoing treatment with these medications. No prior suicide attempts were reported, and no suicidal note was found at the scene or among his belongings. On the outer part of his desk there was a razor blade with a small amount of blood on it. During the previous month, he had been hospitalized for a panic crisis and was discharged one week prior to his body discovery. He was last seen alive by his parents two days prior to his discovery. At the scene, postmortem lividity was observed as confluent on the front side of his face and body, with multiple petechiae in the lividity-dependent areas. Rigor mortis was present throughout the body and the time of death was estimated 18–24 h prior to discovery.

**Fig. 2:**
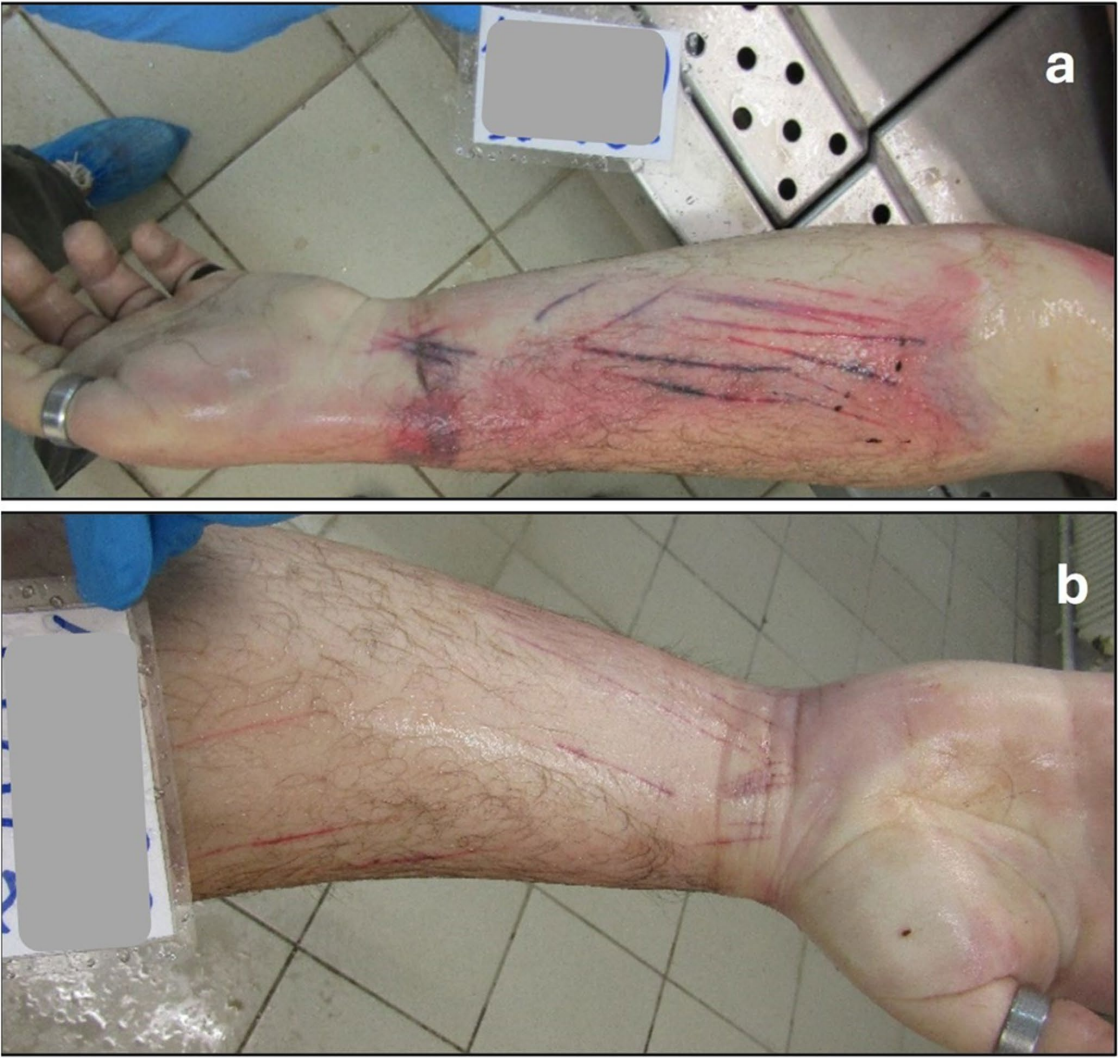
(**a**, **b**): Cutmarks/superficial incisions on the inner surface of left (**a**) and right (**b**) forearms and wrists, respectively (more on the left wrist– right-handed person), running towards different directions, recognized as hesitation marks, corresponding to sharp force injury most likely caused by the razor in Fig. 1

The body was transferred to the mortuary of the Department of Forensic Medicine and Toxicology, Faculty of Medicine, University of Ioannina, Greece, eight hours after discovery and was preserved in the mortuary refrigeration chamber at 2–40 ºC until autopsy, which was performed 12 h post-transfer. During the postmortem examination, as could be seen in Fig. 2, multiple (more than ten) fresh, linear, superficial incisions, of 10 cm maximum length, were observed on the inner surface of both his lower forearms and wrists (more on his left wrist– of note, the decedent was right-handed according to the police information), running obliquely and towards different directions, suggesting hesitation marks caused by a razor blade. No other injuries or lesions were noted.

On internal examination, cerebral edema, lung congestion and pulmonary edema were noted. His heart weighed 450 g and presented no anatomical abnormalities, signs of atherosclerosis or myocardial fibrosis. His stomach contained 250 mL of partially digested food mixed with semi-liquid fluids, and gastric mucosa showed early putrefactive changes. The urinary bladder was markedly distended, containing 600 mL clear urine. The prostate gland was normal both in size and composition. All organs showed early putrefactive changes, with no other macroscopic pathological lesions present.

Histological examination was not performed, as the evident putrefactive processes, would yield only vague findings on histology. Blood samples (peripheral blood from the left external iliac vein), along with urine and gastric contents samples were proceeded for toxicological analysis.

### Toxicological investigation

The autopsy blood specimen was collected in a 2 mL tube, BD Vacutainer^®^ (Becton Dickinson) with presence of anticoagulant and preservative (sodium fluoride 5 mg and potassium oxalate 4 mg). The urine and stomach contents specimens were stored in 120 mL plastic sterile containers (Medimel P.P.C). All specimens were maintained at 4 °C up to two weeks after sample collection when all analyses were completed and then were stored at −20 °C up to six months (in case that re-analyses were requested).

Ethanol and volatiles analysis were performed in whole blood by head space gas chromatography flame ionization (HS-GC-FID) detection [19]. Screening for opiates, benzodiazepines, amphetamines, cannabinoids and cocaine metabolites, in urine and blood, was performed by immunoassays (SYVA, Abbott Park, IL). Toxicological screening for the presence of common drugs and poisons was carried out by a routine gas chromatography–mass spectrometry (GC–MS) technique in full-scan mode on whole blood and urine extracts, following solid phase extraction (Chem Elut cartridges, Agilent Technologies, Lake Forest, CA, USA). The mass spectral libraries used for identification of compounds were the NIST27 and PMW-tox3. GC–MS analyses were performed using a Shimadzu GC17A-QP5050 GC–MS instrument equipped with an Equity5 column (30 m × 25 mm id), 95% dimethyl-5% diphenylpolysiloxane, film thickness 0.25 μm, purchased from Supelco (Bellefonte, PA). Helium was employed as the carrier gas with a constant flow rate at 1.5 mL/min. For the screening analysis the GC oven temperature was programmed to rise from 60° to 280 °C using a step-temperature program and the total run time was 36.17 min. The GC injector and transfer line were maintained at 260 °C and the injector was operated in the split less mode. Full scan spectra were acquired in the interval 40–550 amu operating in the EI mode at 70 eV. Paroxetine levels in blood were determined by a liquid chromatography–mass spectrometry (LC-MS/MS) method [20], properly modified to apply for urine and gastric contents analysis. Aliquots of 200 µL of whole blood or urine, 200 µL of carbonate buffer (1 M, pH 9.5), and 20 µL of deuterated drugs mixture were extracted with of 1.0 mL methyl tert-butyl ether (MTBE)/0.1 M HCL (37%), stirred, centrifuged and the supernatant was evaporated to dryness with a current of N_2_, at a temperature of 40 °C. Reconstitution was performed in 50 µL of solvent A: B (88:12%), and samples were transferred to autosampler vials for LC-MS/MS analysis. Analyses were performed in triplicate. Gastric contents of about 1 mL were diluted with 2 mL double distilled water, 2 drops of NaOH, 5 M were added and vortex followed. The mixture was extracted with 3 mL of diethyl ether, vortexed and centrifuged (20 min, 4000 rpm). The supernatant was collected, evaporated and re-dissolved in 50 µL A: B (88:12%), stirred for 30 s and injected into the LC-MS/MS system.

LC-MS/MS instrumentation was a Dionex UltiMate 3000 UHPLC system (Thermo Scientific, Waltham, MA, USA) comprised of a binary pump, coupled to a Q-Trap 5500+™ mass spectrometer (Sciex, Darmstadt, Germany), operated in multiple reaction monitoring (MRM) mode, and equipped with an electrospray ionization (ESI) Turbo V Source in positive mode. The applied ESI inlet conditions were as follows: gas 1, nitrogen (55 psi); gas 2, nitrogen (55 psi); ion-spray voltage of 5500.0 V, positive mode; ion-source temperature, of 550 °C; nitrogen as the curtain gas at 20 psi. Separation was performed on an Accupore C18 column (50 mm × 3 mm, 2.6 μm particle size) equipped with a precolumn cartridge (2.1 mm × 0.2 μm) (Thermo Scientific, Waltham, MA, USA), both operated at 30 °C. Mobile phases were: 10 mM of aqueous ammonium acetate adjusted to pH 3.5 with 0.1% formic acid (eluent A) and acetonitrile UHPLC-MS grade with 0.1% formic acid (eluent B), degassed by Elmasonic S ultrasonic, Germany. The autosampler temperature was 5 °C.

## Results

The autopsy revealed no external traumatic injuries or internal pathologies. Toxicology showed negative blood ethanol analysis, and no illicit drugs in the analyzed samples. The toxicological screening identified the antidepressant, paroxetine in whole blood, urine, and gastric contents, and detected quetiapine, alprazolam and its metabolite in whole blood and urine. In Table 1, are presented the concentration of the detected drugs. Blood paroxetine levels were higher than the therapeutic levels [12, 13, 21] and within the toxic concentration range that were detected in other paroxetine overdose fatalities [8–11, 22–24]. The concentration of alprazolam in whole blood was within the respective therapeutic range and quetiapine levels were subtherapeutic (Table 1) [12, 13]. Nor-clozapine was detected in urine. Paroxetine, alprazolam, its metabolite alpha-hydroxy-alprazolam, and quetiapine were detected qualitatively in gastric contents, as well.

**Table 1:** Drug concentrations detected in the postmortem whole blood and urine of the presented case; Paroxetine plasma therapeutic levels and paroxetine concentrations detected in overdose fatalities are listed for comparison

Drugs, µg/mL	Blood*	Urine	Therapeutic levels, serum	Fatal Cases/Whole Blood	Ref.
Paroxetine	3.3	1.6	< 0.25	0.176–3.2/**Femoral**	[8, 22, 23]
				1.4–4.0/**Heart**	[8, 11–13, 20, 24]
				1.58/**Subclavian**	[9]
				0.4/**Peripheral**	[10]
*Quetiapine*	0.0013	0.0076	0.025–0.365	0.95–12.7	[12, 13]
*Alprazolam*	0.038	0.495	0.005–0.050	0.3 <	[12, 13]
*a-OH-Alprazolam*	0.0056	0.190			
*Norclozapine*	-	0.0165			

* Collected from the external left iliac vein

## Discussion

This is a case report describing the death of a 22-year-old man attributed to the intentional overdose of the antidepressant paroxetine. All evidence, including the findings at the scene by the police, the autopsy performed by the forensic pathologist, and the toxicological results, were consistent with suicide as the manner of death. The traumatic lesions on his arm corresponded to self-injuries, indicative of a previous attempt to commit suicide. The empty scattered packets of medicines indicated their consumption by the decedent. Although there was not a suicide note, the evidence gathered pointed toward suicide.

The toxicological results revealed the presence of lethal paroxetine concentration in the examined postmortem blood as well as the presence of alprazolam at therapeutic levels, its metabolite alpha-hydroxy-alprazolam and quetiapine at sub-therapeutic levels, respectively. The detection of norclozapine in the urine indicated that the deceased had ingested clozapine within a few days prior to death, possibly during his hospitalization about one week prior to his body discovery (clozapine’s mean elimination half time ranges from 8 to 12 h, depending on the dose and the duration of use [13]). The detected postmortem whole blood concentration of paroxetine fell within the range of concentrations reported for postmortem whole blood in acute lethal overdose situations being 0.176–4.0 µg/mL [8–11, 22–24],. Interestingly, in a fatal poisoning case of severe serotonin syndrome and acute respiratory distress syndrome sequential serum concentrations of paroxetine were reported, being 5.38 µg/mL at admission and 3.21 µg/mL on day 7, while the patient died after 23 days due to respiratory failure [25]. The wide range of lethal paroxetine concentrations reported in related fatalities indicates that other factors, such as the concurrent medication or altered pharmacokinetics due to overdose, could significantly impact on the fatal outcome of a certain case [1–11, 14, 22–25].

The possibility of QT interval prolongation due to paroxetine (an SSRI), which is usually associated with a ventricular arrhythmia that can cause sudden cardiac death [6] could not be excluded nor documented as a mechanism of death in the current case. Additionally, we cannot exclude or document serotonin syndrome [7] as the mechanism that resulted to the current death. It is worth mentioning that in previous fatalities due to paroxetine overdose serotonin syndrome has been considered as the main contributor to death [8–10]. Moreover, the possibility of ventricular arrhythmia exerting complex vasoactive effects on the vascular smooth muscle [10] also could not be excluded nor be documented as a possible mechanism of toxicity.

Finally, paroxetine is subjected to extended postmortem redistribution [13, 22, 23] since a more than fourfold difference in paroxetine concentration between heart and femoral blood (0.78 and 3.20 ng/mL, respectively) was reported [8, 22]. Although the collection site in the current case was peripheral (as it is suggested to minimize the impact of postmortem redistribution), the significant putrefaction of the body could have affected the drug concentration positively or negatively [26].

It is worth mentioning that paroxetine, like other SSRIs, may increase the risk of suicidal ideation and suicidality among individuals under the age of 25 years old [18]. Furthermore, the current case is in line with the reported high risk of suicides and attempts soon after psychiatric hospital discharge, with one-third of the one-year risk occurring within the initial two weeks [16] and the frequent use of psychiatric medication for suicides [27]. These facts underline the importance of pharmaco-surveillance, especially for psychiatric patients.

In conclusion, the manner and cause of death in this case was recorded as suicide and, mixed-drug intoxication due to the synergistic action of the primary toxicant paroxetine and the secondary contribution of alprazolam, quetiapine according to the complete medicolegal investigation that was performed, including the autopsy, the scene investigation, the circumstances of death by the police, the medical history of the decedent, and the toxicological results. To ensure statistical validity in terms of cause and manner of death, we aligned our findings with the hierarchy underlined in the International Classification of Diseases (10th revision) and it was concluded that death was classified as X61. This code stands for “Intentional self-poisoning by and exposure to antiepileptic, sedative-hypnotic, antiparkinsonism and psychotropic drugs, not elsewhere classified”.
